# Clinical Impact of CD25/CD123 Coexpression in Adult B-Cell Acute Lymphoblastic Leukemia Patients

**DOI:** 10.1155/2020/9545717

**Published:** 2020-05-20

**Authors:** Salah Aref, Mohamed El Agdar, Nada Khaled, Lamyaa Ibrahim, Mohamed S. El-Ghonemy

**Affiliations:** Hematology Unit, Clinical Pathology Department, Oncology Center Mansoura University (OCMU), Mansoura University, Mansoura, Egypt

## Abstract

This study aimed to determine the clinical impact of CD25^+^/CD123^+^ coexpression in adult B-cell acute lymphoblastic leukemia (B-ALL) cases. One hundred and twenty newly diagnosed B-ALL patients (≤60 years old) were included in this study. CD123 and CD25 expression on leukemic blast cells were assessed using flow cytometry. CD25^+^/CD123^+^ coexpression was detected in 40/120 B-ALL patients (33.3%). All B-ALL patients showed CD25^+^/CD123^+^ coexpression had lower induction of remission response and shorter overall survival as compared to B-ALL cases lacking coexpression. In conclusion, CD25^+^/CD123^+^ positive coexpression is a reliable flow cytometry marker for prediction of the outcome of adult B-ALL patients and could be used as a novel parameter for risk stratification of adult B-ALL cases.

## 1. Introduction

Acute lymphoblastic leukemia (ALL) is a neoplastic disease characterized by clonal expansion of leukemic cells in the bone marrow (BM), lymph nodes, thymus, or spleen. It is a heterogeneous disease characterized by multiple subtypes [1].

Treatment strategies of adult B-ALL patients are based on various prognostic factors, including age and performance status of the patient, as well as cytogenetic and molecular characteristics of the leukemic clone [2–5].

Philadelphia positive (Ph^+^) B-ALL accounts for 3–5% in children and 20–30% in adults, and the incidence increases to about 50% in patients aged >50 years. Patients with Ph^+^ and Ph^+^-like molecular and cytogenetic signatures were frequently associated with adverse prognosis before the era of targeted treatment using a tyrosine kinase inhibitor (TKI) in combination with conventional chemotherapy, which has dramatically improved the outcome of this previously poor prognostic group [6]. The cytogenetic and molecular abnormalities are not present in all B-ALL cases [7].

Current research focuses on the detection of novel prognostic markers that could predict the outcome of B-ALL patients. Several studies have reported correlations of leukemia-associated markers with cytogenetic findings and clinical outcome in B-ALL patients. These include cluster of differentiation (CD)-25 (CD25) and interleukin-3 receptor alpha chain (IL-3R*α* (CD123)) [6, 8, 9].

CD25 represents the *α*-chain of the interleukin-2 receptor (IL-2R*α*), which is a low-affinity binding receptor. The IL-2 receptor is composed of different combinations of three subunits (alpha, beta, and gamma chains) and is normally expressed on activated T-cells. Upon binding its ligand IL-2, the IL-2 receptor induces T-cell proliferation and differentiation [10]. The expression of CD25 by flow cytometry (FC) has been described as a poor prognostic factor in ALL, and it was suggested to be a valuable biomarker to identify a subset of patients with Ph^+^ who would benefit from a TKI-based chemotherapy [6, 10].

CD123 is expressed in several hematologic neoplasms, including B-cell ALL, but expressed at a low level or to be absent on normal hematopoietic stem cells [8]. Importantly, it has been reported that CD123-positive expression was detected in leukemic stem cells and at more differentiated leukemic blast cells [9, 11].

This study aimed to assess the pattern of CD25/CD123 expression and its clinical value in adult B-ALL patients.

## 2. Methods

One hundred and twenty newly diagnosed adult B-ALL patients ≤60 years old before the start of therapy were included in this study. All included patients gave informed consent. Bone marrow and/or peripheral blood samples were taken from the B-ALL patients at the time of diagnosis. The patients were subjected to morphologic examinations of both the peripheral blood smear and bone marrow smear (blast cells ≥ 20%). Immunophenotyping evaluation was done using the following combination of monoclonal antibodies (MoAbs) panels for acute leukemia diagnosis and to identify B-ALL subtypes: CD34.PE (CLONE 8G12 BD), TDT.PE (CLONE E17-1519 BD), anti-MPO.FITC (CLONE CLB-MPO-1 BC), CD45.APC (CLONE H130 BD), CD79a.PE (CLONE HM47 BD), CD20.FITC (CLONE B9E9 BC), CD10.PE (CLONE H110a BD), CD19.APC(CLONE J3-119 BC), CD22.PC 5.5 (clone SJ10. 1H11 BC), CD7.PE (CLONE M-T701 BD), CD2.FITC (CLONE RPA-2. 10 BC), CD3.PC 5.5 dye (clone UCHT1 BC), CD117.PE (clone 104D2D1 BC), CD33.PE (CLONE WM53BD BD), CD13.PE (CLONE WM15 BD), CD14.PE (clone M5E2 BD), and CD64.FITC (CLONE MA-251 BD PHARMAGEN). All antibodies were purchased from BD Biosciences and Beckman Coulter (BC). Samples were analyzed on a BD FACS Canto™ flow cytometer. Autofluorescence, viability, and isotype controls were included. Flow cytometric data acquisition and analysis were conducted by BD Cell Quest™ Pro software.

The cytogenetic evaluation was performed using FISH techniques for detection of Philadelphia chromosome (BCR/ABL fusion gene). The patients were followed up for 24 months or until death. The study has been approved by the Mansoura Faculty of Medicine Local Ethics Committee and that it conforms to the provisions of the Declaration of Helsinki.

### 2.1. Flow Cytometric Determination of CD25/CD123 Cell Antigen Expression

For CD25 and CD123 analysis, the stain/lyse/wash technique was used. Briefly, in single tube, 10 *μ*l of the CD25-PE MoAb (CLONE MA-251 BD PHARMAGEN), 10 *μ*l of CD123-FITC MoAb (CLONE 7G3 BD PHARMAGEN), and 10 *μ*l of CD45 APC (CLONE H130 BD PHARMAGEN) were added to 100 *μ*l of ethylenediaminetetraacetic acid (EDTA) fresh bone marrow samples, mixed well, and incubated for 15 minutes at room temperature. The cells were then washed twice with phosphate-buffered saline (PBS); 2 ml lysing solution was added, mixed, and left for 15 minutes in the dark, and then the cells were washed twice with PBS. After the last wash, the cells were suspended in 500 *μ*l of PBS, and then analyzed using a flow cytometer (FACSCanto flow cytometer with CellQuest software; Becton Dickinson) [12]. At least 10,000 events/tube were measured.

The blast gate was defined based on CD45 dim expression and side-scatter characteristics and calculated as a percentage of total gated events. For analysis of CD123 expression, measurements included mean fluorescence intensity (MFI) on leukemic blasts (adjusted for background fluorescence using negative internal controls) and relative mean fluorescence intensity (RFI) ratio (divide the MFI values of defined leukemic blasts by nonleukemic events). In patients' samples, CD25 or CD123 was defined as a positive expression if ≥20% of leukemic blasts using MFI in excess of the background fluorescence in the negative controls (nonleukemic gated events) [13].

### 2.2. Statistical Analysis

Patient data were compared by the use of the Fisher exact test if they were categorical (qualitative data, presented as number and percent) and the Wilcoxon rank-sum tests if they were continuous (quantitative data, presented as mean ± SD).

Overall survival (OS) was defined as the time from randomization to death from any cause. OS probabilities were estimated by the use of the Kaplan–Meier method, and statistical significance of associations was assessed with the log-rank test.

Antigen expression data are described by the use of descriptive statistics of observed values. The percentages of antigen-expressing blast cells for CD25/CD123 were considered continuous variables and compared with the nonparametric Wilcoxon rank-sum test.

## 3. Results

The patterns of CD25 and CD123 expression in B-ALL cases are presented in Table 1. Positive expression was considered at cutoff  ≥ 20%. The B-ALL patients who showed CD25^+^/CD123^+^ expression was categorized as group 1, while those who showed single-positive expression (CD25^+^/CD123^−^ or CD25^−^/CD123^+^) and double-negative expression (CD25^−^/CD123^−^) were categorized as group 2. CD25^+^/CD123^+^ coexpression was detected in 40/120 (33.3%) B-ALL cases. Single-positive (CD25^+^/C123^−^ and CD25^−^/CD123^+^) expression was detected in 25/120 (20.8%) and 20/120(16.7%), respectively. Double-negative (CD25^−^/CD123^−^) expression was detected in 25/120 (20.8%) B-ALL cases.

The impact of CD25/CD123 expression pattern on B-ALL patient's characteristics is shown in Table 2. The pattern of expression was categorized into 2 groups. Group 1 included cases which showed CD25^+^/CD123^+^ coexpression, while group 2 included cases which showed CD25^−^/CD123^−^ plus CD25^+^CD123^−^ and CD25^−^/CD123^+^ expressions. Group 1 (CD25^+^/CD123^+^) has higher incidence of CNS infiltration and lymphadenopathy, lower rate of induction remission response, and a higher number of Ph^+^, but lower concentration levels of serum LDH as compared to group 2 (single positive and double negative for CD25/CD123) (*p* = =0.01; 0.05; <0.001; <0.01, respectively). The remaining parameters did not significantly differ in the 2 groups (*P* > 0.05).

Using the FISH technique, the Philadelphia chromosome was detected as positive in 36 out of 120 (30%) B-ALL patients. Most of the B-ALL patients (36/40; 90%) who showed CD25^+^/CD123^+^ coexpression were Philadelphia chromosome positive (Table 3).

**Figure 1:**
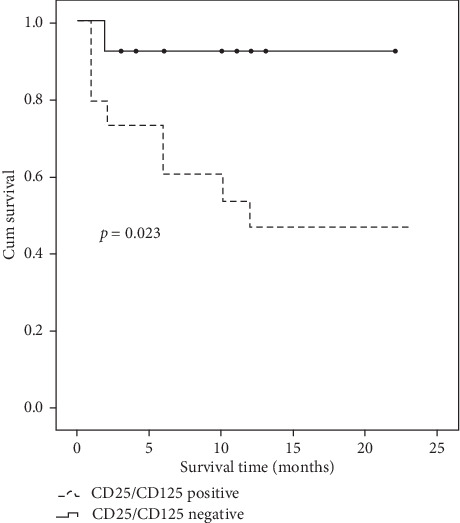
Survival curve for adult B-ALL CD25^+^/CD123^+^ (double-positive) cases vs those with CD25 or CD123 (single-positive) or CD25^−^/CD123^−^ (double-negative) ones.

The effect of CD25/CD123 expression on the B-ALL patient overall survival (OS) revealed that adult B-ALL patients who showed CD25^+^/CD123^+^ coexpression (double-positive) had significantly shorter OS as compared to the negative ones (single positive and double negative) (Table 4). Survival analysis studies using the Kaplan–Meier curve revealed that B-ALL patients who showed CD25^+^/CD123^+^ coexpression had shorter OS as compared to the negative ones (*p* < 0.01) (Figure 1).

## 4. Discussion

In recent years, emerging research has found that CD123 and CD25 are highly expressed on the surface of various leukemic blasts cells and may contribute to the proliferative advantage of leukemic cells. As a novel biological marker, it has an attractive prospect in the diagnosis, targeted therapy, and evaluation of prognosis of many diseases [14].

In the current study, CD25^+^/CD123^+^ coexpression was detected in 33.3% (40 out of 120) of the investigated B-ALL patients. To the best of our knowledge, no previous study has reported the CD25^+^/CD123^+^ coexpression in such a cohort of patients.

CD25+ expression was detected in 53.8% (33.3% in the double-positive group and 20.5% in the single-positive group). This figure is higher than that detected by Nakase et al. [10] (25%), Jaso et al. [15] (30%), and Owaidah et al. [16] (32%). This disagreement could be attributed to the higher cutoff value (30%) considered by previous studies to confirm CD25+ expression.

CD123^+^expression was found in 50% of the studied B-ALL patients (33.3% in the double-positive CD25^+^/CD123^+^ group plus 16.7% in the single-positive CD25^−^/CD123^+^ group). This figure is lower than that reported by Angelova et al. [8] (164/183 (89.6%)); Djokic et al. [17] detected 31% strong expression, 61% moderate expression, and 8% negative, and Bras et al. [18] who detected CD123^+^ expression in 85% of B-cell precursor (BCP) ALL cases (224/262). These controversies could be explained on the basis of the difference in the cutoff value used for detection of the CD123^+^ expression in different studies.

In the current study, Ph^+^ was detected in 30% (36/120) of B-ALL cases. This finding is nearly similar to that reported by Gadhia et al. [19] (33.3%) and higher than that reported by Obadiah et al. [16] (17.5% (18/103)).

In the present study, the median serum LDH levels were significantly higher in patients lacking CD25^+^/CD123^+^ coexpression as compared to those who showed CD25^+^ or CD123^+^ single expression. This finding could be explained on the basis that B-ALL patients group lacking CD25^+^/CD123^+^ coexpression has higher median blast cells counts as compared to those with CD25^+^/CD123^+^ positive coexpression. A previous report stated that increased values of serum LDH directly reflect the tumor mass in patients with ALL [20].

In order to assess the prognostic relevance of CD25^+^/CD123^+^ coexpression, we have correlated this expression with a well-known prognostic factor which is the Philadelphia chromosome. We found strong association between CD25^+^/CD123^+^ positive coexpression and Ph^+^ patients (*p* < 0.01). All Ph^+^ patients showed CD25^+^/CD123^+^ coexpression. In parallel with our finding,Angelova et al. [8] reported that CD123 expression was more prevalent in Ph^+^ patients than in Ph^−^patients (96.6% versus 86.3%; *p* = 0.033).

BCR/ABL was detected in 36 out of 120 (30%) B-ALL cases. This finding is slightly higher than that detected previously which was 22% [10, 15]. Moreover, in these two previous studies, they found an association between CD25 expression and Ph^+^ in adult B-ALL cases. Likewise, Owaidah et al. [16] and Gaikwad et al. [21] found that all BCR/ABL-positive cases were positive for surface CD25. Furthermore, Chen et al. [6] reported that CD25 expression (using 15% as a cutoff) in B-ALL predicts Ph^+^ (80% sensitivity, 86% specificity, 37% positive predictive value, and 97% negative predictive value). Lastly, Gönen et al. [22] reported that CD25^+^ expression is an independent predictor of the outcome of acute myeloid leukemia patients.

B-ALL patients with CD25^+^/CD123^+^ coexpression showed lower induction of remission rate and shorter overall survival as compared to negative ones. These results could be explained on the basis that overexpression of CD123 defines a subset of blast cells which are resistant to chemotherapy and seems associated with high relapse rate [14, 23].

In conclusion, positive CD25^+^/CD123^+^ positive coexpression defines a subset of B-ALL patients with poor outcome and could be helpful to refine the risk stratification of B-ALL cases at diagnosis.

## Figures and Tables

**Table 1:** The pattern of CD25 and CD123 expression in adult B-ALL cases.

	Adult B-ALL (*n* = 120)
CD25^+^/CD123^+^ (double positive)	40/120 (33.3%)
CD25^+^/CD123^−^ (single positive)	25/120 (20.8%)
CD25^−^/CD123^+^ (single positive)	20/120 (16.7%)
CD25^**−**^/CD123^−^ (double negative)	25/120 (20.8)

**Table 2:** Characteristics of CD25^+/^CD123^+^ double positive in adult B-ALL cases in comparison with CD25/CD123 single positive and double negative.

Parameters	CD25^+^/CD123^+^ coexpression Adult B-ALL cases (group 1) (*n* = 40)	CD25 or CD123 Single positive or double negative (group 2) Adult B-ALL cases (*n* = 80)	*p* value
Age median (range)	42 (16–64)	44 (17–59)	>0.05
Male sex, *n* (%)	9 (45%)	21 (52.5%)	>0.05
Hb g/dl median (range)	9.2 (4.1–12.0)	9.0 (3.9–12.8)	>0.05
WBCs × 10^9^/L median (range)	34.2 (2.2–123.0)	31.0 (1.4–140)	>0.05
Platelets × 10^9^/L median (range)	53.0 (4.0–76.0)	50.0 (4.0–74.0)	>0.05
Blood blasts % median (range)	48 (0–96)	52 (0–98%)	>0.05
BM blasts % median (range)	88 (44–99)	90 (42–100)	>0.05
Serum LDH IU/L median (range)	1012 (420–21800)	1230 (288–11012)	0.001
CNS infiltration, *n* (%)	10 (30%)	4 (5%)	0.01
Lymphadenopathy, *n* (%)	6 (15%)	10 (12.5%)	0.05
Hepatomegaly, *n* (%)	2 (5%)	6 (7.5%)	>0.05
Splenomegaly, *n* (%)	8 (20%)	14 (17.5%)	>0.05
Induction of remission response, *n* (%)	20 (50%)	72 (90%)	<0.001
Cytogenetic BCR-ABL (positive)	36/40	0/80	<0.01

**Table 3:** Association between CD25^+^/CD123^+^ coexpression and Philadelphia (Ph^+^) (BCR/ABL) in adult B-ALL cases.

B-ALL cases (*n* = 120)	Ph^+^ B-ALL cases (*n* = 36)	Ph^−^ B-ALL cases (*n* = 84)	*p* value
CD25^+^/CD123^+^ coexpression B-ALL cases (group 1) (*n* = 40)	36	4	<0.01
CD25 or CD123 Single positive or double negative Adult B-ALL cases (group 2) (*n* = 80)	0	80	

**Table 4:** Impact of CD25^+^/CD123^+^ coexpression on the outcome of adult B-ALL cases.

B-ALL cases (*n* = 120)	CD25^+^/CD123^+^ coexpression B-ALL cases (*n* = 40)	CD25 or CD123 Single positive or double negative Adult B-ALL cases (*n* = 80)	*p* value
Survivors	18	72	<0.01
Deaths	22	8	

## Data Availability

The data used to support the findings of this study are available and may be released upon application to the corresponding author.
